# Prognostic significance of E-Cadherin and CD44 expression in tumor tissue of colorectal carcinoma

**DOI:** 10.1186/1753-6561-9-S1-A10

**Published:** 2015-01-14

**Authors:** T Knezevic, L Kosi, I Kadic

**Affiliations:** 1 School of Medicine, University of Belgrade, Belgrade, Serbia

## Background

Colorectal cancer (CRC) is a major cause of cancer-related mortality worldwide. Currently, tumor-node-metastasis staging system still remains the gold standard for prognosis of CRC patients, but it is no longer sufficient. Among the most important molecules involved in colorectal tumor growth, cell proliferation and progression are adhesive molecules such as CD44 and E-cadherin (EC). The aim of this study was to evaluete the expression of CD44 standard protein and EC in correlation with Ki-67 proliferative index and some other clinicopathological prognostic variables in patients with CRC.

## Methods

Eighty one patients with CRC were studied by hematoxylin-eosin stain on formalin fixed paraffin embedded tumor tissue obtained by radical surgery. Ki-67, EC and CD44 were analysed by immunohistochemistry, using labeled-streptavidin-biotin (LSAB) method with anti Ki-67, anti-EC and anti-CD44 standard monoclonal antibodies in optimal concentrations. of immunohistochemical analysis were evaluated semi-quantitatively by determination of percent of CD44 positive cells and presence/abssence of EC positive cells. Ki-67 proliferative index was estimated as percent of positive cells by counting 500 tumor cells were analysed using statistical tests, adopting a significance level of 5%.

## Results

36 of the 81 analyzed patients were in stadium C and D according Dukes original classification, 24 in stadium B and 21 in stadium A. Increased CD44 expression was present in 87.7% of patients with disease progression versus 12.3% of patients without progression (p≤0.05). Overall survival in patients with high CD44 expression was 23.5 months versus 35 months in the low CD44 expression group (p≤0.05). Monitoring of desease progression within two years period and overall survival showed a statistically significant difference between the group of patients with heterogenous or loss of EC expression and group with regular EC expression (78.3% and 21 months versus 47.6% and 46 months respectively). However, there were no significant differences in the Ki-67 proliferative index between the group with high CD44 and heterogeneous or loss of E-cadherin expressin and group with low CD44 and regular E-cadherin expression.

## Conclusions

The investigated molecular markers may be useful for the prediction of outcomes and rate of recurrence in CRC patients who have been operated on. In conjunction with clinical and pathological staging, they may provide a stronger indication of clinical outcomes than staging alone and help improve selection of therapeutic options in CRC patients.

